# Farletuzumab ecteribulin and MORAb‐109, folate receptor alpha and mesothelin targeting antibody–drug conjugates, show activity in poor prognosis gynaecological cancer models

**DOI:** 10.1002/ctm2.70274

**Published:** 2025-03-12

**Authors:** Ksenija Nesic, Katherine Rybinski, Gayanie Ratnayake, Gwo‐Yaw Ho, Ratana Lim, Marc Radke, Chloe Neagle, Elizabeth M. Swisher, Matthew J. Wakefield, Holly E. Barker, Keiji Furuuchi, Clare L. Scott, Cassandra J. Vandenberg

**Affiliations:** ^1^ Walter and Eliza Hall Institute of Medical Research Parkville Victoria Australia; ^2^ The University of Melbourne Parkville Victoria Australia; ^3^ Eisai Inc Exton Pennsylvania USA; ^4^ The Royal Women's Hospital Parkville Victoria Australia; ^5^ School of Clinical Sciences, Monash University Clayton Victoria Australia; ^6^ University of Washington Seattle Washington USA; ^7^ Department of Oncology Sir Peter MacCallum Peter McCallum Cancer Centre Melbourne Victoria Australia

1

Dear Editor,

Few therapeutic options are available for aggressive, poor prognosis gynaecological cancers (GC), including uterine serous carcinoma (USC) and ovarian clear cell adenocarcinoma (OCCA). We observed deep and durable responses to the antibody‐drug conjugates (ADC), farletuzumab ecteribulin (FZEC, previously known as MORAb‐202) or MORAb‐109, in GC patient‐derived xenograft (PDX) models expressing corresponding target antigens, folate receptor alpha (FRA) or mesothelin (MSLN), providing evidence for clinical trial inclusion of these GC types. Resistance was observed in PDX models with high *ABCB1* expression, highlighting this as a potential exclusion criterion in clinical trials of *ABCB1* substrates.

The anti‐microtubule agent (AMA), paclitaxel, is commonly used in first‐line treatment for most GC, in combination with the platinum agent carboplatin, but resistance is common. The AMA, eribulin, has demonstrated efficacy in breast and non‐small cell lung cancers^1^ and we have shown preclinical efficacy in treating aggressive, poor prognosis GC.^2^, ^3^ ADCs have enabled targeted delivery of potent cytotoxic agents, including in GC.^4^, ^5^ Both FRA and MSLN are not generally expressed in normal adult tissues, but given high frequency of expression in ovarian, uterine and other solid cancers, they have become attractive ADC targets.^5^ Here, we investigate the utility of the anti‐FRA‐eribulin ADC, FZEC, and the anti‐MSLN‐eribulin ADC, MORAb‐109, in the treatment of aggressive GC.^6^, ^7^, ^8^

We screened 41 GC PDX models covering 11 poor prognosis GC subtypes to determine the frequency and distribution of FRA and MSLN expression (Table S1). For high‐grade serous ovarian cancer (HGSOC), 14 PDX models were assessed for FRA expression, with 5/7 chemo‐naive and 6/7 post‐treatment models being designated as FRA positive (total FRA > 5%, with various staining patterns described; Figure 1A). Three post‐treatment HGSOC models, reflecting the target patient group for clinical trials, were selected for FZEC treatment (Table S2). To investigate low versus high expression of FRA, the PDX models #111 (6% FRA+), #206 (32% FRA+) and #931 (43% FRA+) were chosen and treated with a 3‐week regimen of eribulin, or with FZEC administered as a *single dose* (Figure 1A; the FZEC single dose equates to 0.25 mg/kg eribulin payload). We observed comparable eribulin and FZEC activity in all three models (Figure 1B, Table S3) and progressive disease (PD) was observed 85 days or more after treatment. We next investigated if repeated dosing with FZEC could extend response duration, testing this in PDX #111, which showed PD at 85 days with the single FZEC dose. Three weeks of weekly dosing produced a complete response (CR; tumour < 50 mm^3^ for ≥3 consecutive weeks) for all mice treated, with no PD by 120 days post‐treatment initiation (Figure 1B, Figure S1 and Table S3).

**FIGURE 1:**
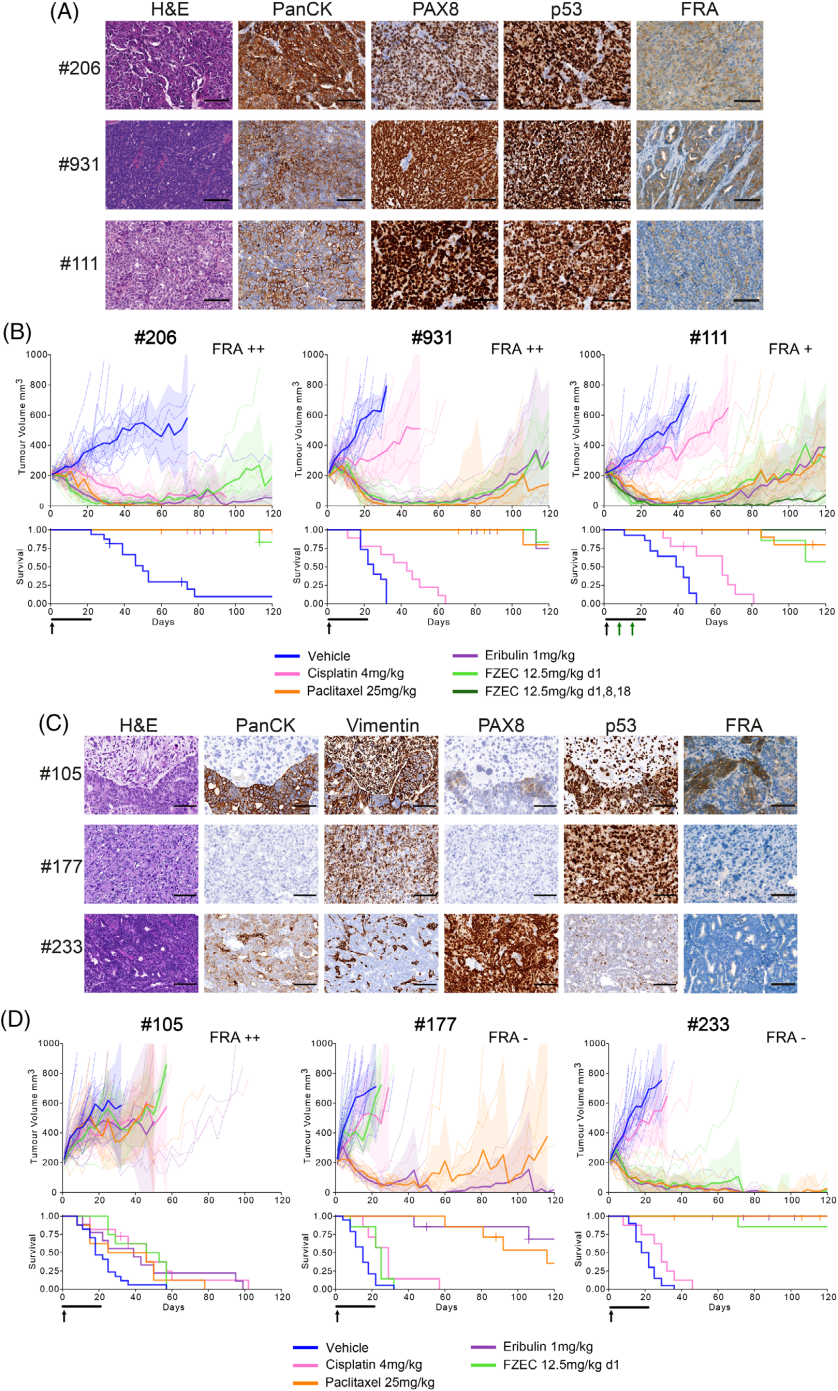
Complete response to a single dose of FZEC in FRA‐positive HGSOC PDX models. (A) Histological assessment of HGSOC PDX models #206, #931 and #111 demonstrate typical staining for pan‐cytokeratin (PanCK) and PAX8 and a high level of p53 expression indicative of mutant *TP53*. FRA IHC was performed using anti‐FRA monoclonal antibody clone 26B3. Staining conditions were selected based on negative staining with an isotype‐matched monoclonal antibody (Ab). FRA expression varies between the PDX models with PDX #206 displaying both apical and membrane staining, while #111 shows apical staining only (for % FRA expression see Table S2). Scale bar represents 100 µm. (B) Response to a *single dose* of FZEC (12.5 mg/kg, intravenous (IV); indicated by the black arrow at day 1 of the Kaplan–Meier survival curves) was compared to a 3‐week eribulin regimen (1 mg/kg, 3 times weekly, intraperitoneal (IP); indicated by the black line below x‐axis) in FRA positive HGSOC PDX models. This dose of FZEC, 12.5 mg/kg, corresponds to only 0.25 mg/kg eribulin when molar mass and drug‐to‐antibody ratio (DAR) of 4 are considered. Additionally, for PDX #111 FZEC repeat dosing was tested (green arrows, day 8 and 18). Response was also compared to the standard HGSOC treatments cisplatin and paclitaxel (cisplatin 4 mg/kg, days 1, 8 and 18, IP; paclitaxel 25 mg/kg, twice weekly, IP). Solid lines on tumour volume curves represent the mean and dotted lines indicate the individual tumours, shaded areas represent the 95% confidence interval. For the number of mice treated, time to PD, median time to harvest (TTH), and log‐rank survival tests see Table S3. The level of FRA expression is indicated on the top right of each graph (+++ indicates > 50% total expression, ++ 25–50%, + 5–25% and— < 5%). (C) Histological assessment of FRA expression in OCS PDX models. OCS tumours are composed of both carcinomatous (epithelial, panCK positive), sarcomatous (mesenchymal, vimentin positive) and mixed (transitional, dual panCK and vimentin positive) components. The composition of OCS PDX models is dependent on the sample of patient tumour from which they are derived. In PDX #105, FRA expression correlated with panCK expression (for % FRA expression see Table S4). Scale bar represents 100 µm. (D) Response to a *single dose* of FZEC (black arrow) was compared to 3‐week treatment regimens for eribulin and the standard OCS treatments cisplatin and paclitaxel (dose and regimen as for (B) above). Only the FZEC response of PDX #177 is as predicted by FRA expression. PDX #105 is resistant to all treatments and PDX #233 is extremely sensitive to all AMAs. For the number of mice treated, time to PD, median TTH, and log‐rank survival tests see Table S3.

Eribulin has shown promising preclinical results in ovarian carcinosarcoma (OCS),^2^ therefore we assessed whether FZEC may offer an effective therapeutic avenue for this rare and aggressive cancer. OCS vary in their carcinoma/sarcoma composition, seen morphologically and by pan‐cytokeratin and vimentin staining^2^ PDX #177, with FRA expression < 1%, was purely sarcomatous (Figure 1C, Table S4). PDX #105 and #233 both had mixed composition: for PDX #105, FRA expression was mainly observed in the carcinoma component (47% FRA+), for PDX #233 although predominantly carcinomatous in appearance, it had < 1% FRA positivity (Figure 1C, Table S4). Despite high FRA expression, PDX #105 was resistant to FZEC, as it also was to eribulin, and standard chemotherapies (Figure 1D). While PDX #177 was sensitive to eribulin (PD > 120 days), no tumour regression was observed with FZEC, consistent with the lack of FRA expression. Somewhat surprisingly, PDX #233 was equally sensitive to eribulin and FZEC with deep and durable responses (eribulin CR 7/8; FZEC CR 6/7; Figure 1D; Table S3), despite very low FRA positivity. Extreme eribulin sensitivity in PDX #233 may drive FZEC response via bystander effect, despite low target expression. FZEC binding by Fc/C‐type lectin receptors on macrophages or neutrophils in the tumour microenvironment can also lead to internalisation, ADC cleavage and release of eribulin.^4^

Of the additional 27 non‐HGSOC GC PDX models assessed, total FRA expression of ≥5% was seen in 13/27 (48%) and total MSLN expression of ≥5%, in 6/27 (22%) (Table S1 and Table S5). FRA expression was more common in epithelial cancers, OCCA (3/3) and USC (6/8). MSLN expression was not as frequent, however, certain PDX models expressed MSLN, without expressing FRA. Nine PDX models were selected for comparison of eribulin versus FZEC versus MORAb‐109, with priority given to models with dual FRA and MSLN expression—three OCCA and six USC PDX models (Table S5, Figure S2). The dual FRA and MSLN expressing models were USC #33, USC #256, OCCA #108 and OCCA #279. PDX #256 had deep and durable responses to eribulin, FZEC and MORAb‐109 (CR in 85%–100%; Figure 2 and Table S6). PDX #33 and #108 had relatively short eribulin responses (time to PD 50 and 67 days respectively), while FZEC and MORAb‐109 significantly extended survival compared to eribulin (Table S6). OCCA PDX #279 was resistant to all treatments despite high FRA and MSLN expression (Figure 2).

**FIGURE 2:**
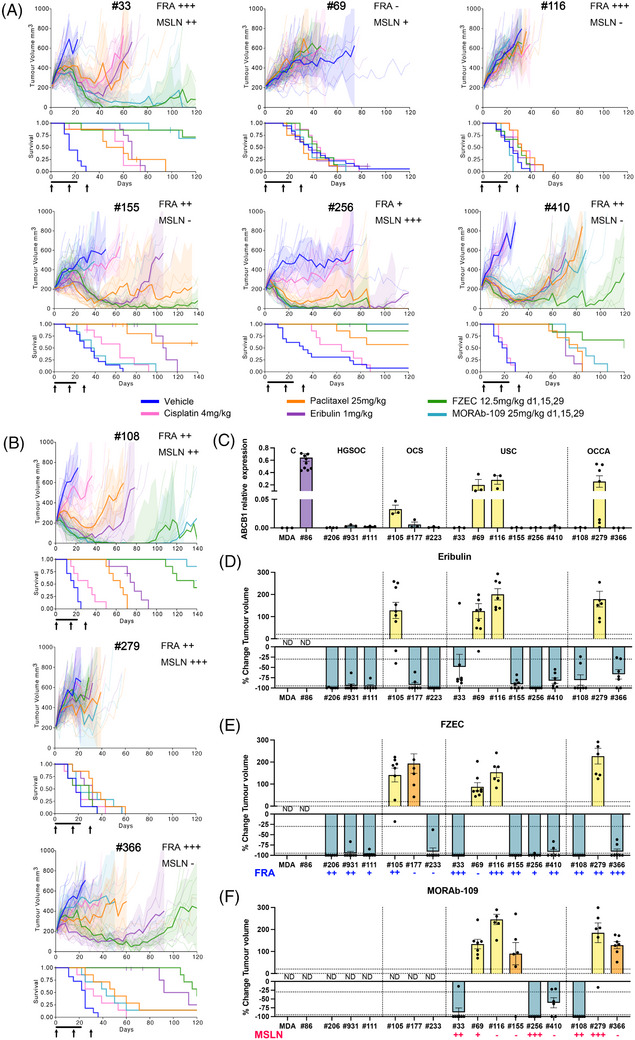
Preclinical models of poor prognosis GC reveal potential for FZEC and MORAb‐109. (A) PDX models of USC were treated with eribulin and the eribulin ADCs FZEC and MORAb‐109. Fortnightly × 3 doses of FZEC (12.5 mg/kg, IV) or MORAb‐109 (25 mg/kg, IV) were administered (indicated by black arrows) and compared to a 3‐week treatment regimen for eribulin (1 mg/kg, 3 times weekly, IP; indicated by the black bar below the *x*‐axis). For FZEC and MORAb‐109, a fortnightly regimen of 3 doses was used to replicate the likely clinical regimen. The DAR of FZEC and MORAb‐109 is 4 and 2, respectively, therefore the doses used were equivalent in terms of eribulin administered. Standard treatments cisplatin and paclitaxel included for comparison (cisplatin 4 mg/kg, day 1, 8 and 18, IP; paclitaxel 25 mg/kg, twice weekly, IP). FRA and MSLN expression are indicated on the top right of each graph (+++ indicates > 50% total expression, ++ 25–50%, + 5–25% and— < 5%). Significantly improved survival compared to eribulin was observed for PDX #33 (FZEC *p* = 0.0029; MORAb‐109 *p *= 0.0001), PDX #155 (FZEC vs. eribulin *p* = 0.0011; FZEC vs MORAb‐109 *p* = 0.0005) and PDX #410 (FZEC vs. eribulin *p* = 0.017, FZEC vs. MORAb‐109 *p* = 0.051) consistent with the target antigen expression in these models (Figure S2 and Table S5). (B) Eribulin, FZEC and MORAb‐109 activity were assessed in PDX models of OCCA. Tumour bearing mice were treated as in (A). For the eribulin responsive models #108 and #366, response to FZEC and MORAb‐109 was in accordance with target antigen expression (Figure S2 and Table S5), and significantly improved survival compared to eribulin was observed for PDX #108 (eribulin vs. FZEC *p* = 0.0003 and eribulin vs. MORAb‐109 *p* = 0.0003). See also Table S6. (C) Expression of the *ABCB1* gene, which encodes P‐glycoprotein 1, was assessed in PDX by quantitative RT‐PCR (qRT‐PCR). *ABCB1* mRNA expression is shown relative to housekeeping genes. HGSOC PDX model #86 was used as a positive control for high expression^3^ and the MDA‐MB‐231 cell line as a negative control. RNA was extracted from at least three independent tumours for each model (n = 3 to 7). Bars represent the mean ± SEM. Ordinary one‐way ANOVA with Dunnett's multiple comparisons test: MDA vs. #116, *p* = 0.0343; MDA vs #279 *p* = 0.0195; (D) Comparison of eribulin response for all PDX models; the best response (after at least 21 days) is shown as the percentage change in tumour volume compared with the tumour volume on day 1. Dotted lines at 10% and ‐30% define the range for stable disease; > 10%, progressive disease; < ‐30%, partial response or < ‐95%, complete response. (E) Comparison of FZEC and (F) MORAb‐109 response for all PDX models. Expression of FRA or MSLN is indicated below the graphs (+++ indicates > 50% total expression, ++ 25–50%, + 5–25% and— < 5%). ND, not determined, these models were not treated with MORAb‐109. Bars represent the mean ± SEM. Yellow bars indicate models with high *ABCB1* (A). Orange bars indicate PDX models with resistance due to no/low expression of the target antigen. The number of mice treated for each PDX model is included in Tables S3 and S6.

For USC PDX #155 and #410, and OCCA PDX #366, expression of FRA was 30–50% but MSLN expression was < 5%. (Table S5). Accordingly, FZEC response was more durable than for eribulin, and no MORAb‐109 activity was observed for PDX #155 and #366 (Figure 2A,B and Table S6). USC PDX models #116, with high (> 50%) FRA expression, and #69 were resistant to all treatments.

Our data are consistent with the previously demonstrated on‐target activity of FZEC and MORAb‐109^7,^ ^9^ as PDX models #177 (FRA negative), #155 (MSLN negative) and #366 (MSLN negative) were sensitive to eribulin but resistant to eribulin‐ADC when the target antigen was not expressed.

In total, four drug resistant PDX models were identified (Figures 1 and 2). Paclitaxel and eribulin are known substrates of P‐glycoprotein 1 (P‐gp; encoded by the *ABCB1* gene) and P‐gp expression correlates with paclitaxel resistance and poor patient outcomes.^10^ Four out of 15 PDX models had elevated *ABCB1* mRNA expression, OCS #105, USC #69, USC #116 and OCCA #279; and all four were resistant to eribulin, as well as to FZEC and MORAb‐109, all with PD as their best response (Figure 2C–F). Notably, these AMA‐resistant PDX had all been derived from samples from patients who had received prior chemotherapeutics, including paclitaxel (Tables 1 and 2). Deriving a suitable diagnostic for transporter over‐expression would be beneficial.^11^

**TABLE 1:** High‐grade serous ovarian cancer (HGSOC) and ovarian carcinosarcoma (OCS) patient‐derived xenograft (PDX) treatment cohort summary.

Tumour type	PDX	Stage at Dx	Age at Dx	Patient treated prior to PDX	Patient TTD from diagnosis (months)	No. of prior systemic therapies	Pre‐treatment	Treatment in Pt subsequent to obtaining PDX	BROCA Molecular characterisation	BROCA panel version	paclitaxel response	eribulin response	% total FRA positive	FZEC response	ABCB1 RT‐qPCR
HGSOC	206	IV	45‐49	YES	44	1	1) Carboplatin and Paclitaxel	1) Olaparib and Cyclophosphamide (PD) 2) Carboplatin and Caelyx (PD) 3) Paclitaxel (PR) 4) Etoposide (PD)	germline BRCA1 NM_007294 c.3817C>T p.Q1273* TP53 NM_000546 c.451C>T p.P151S; rearrangement of BLM NM_000057	BROCA‐HR v8	yes	yes	32	yes	LOW
	111	IV	55‐59	YES	31	4	1) Carboplatin and Paclitaxel (PR) 2) Anastrazole (maintenance) 3) Carboplatin, Paclitaxel, AKT inhibitor (SD) 4) AURK inhibitor (SD) 5) RTx	1) Olaparib and Cyclophosphamide (PD)	CCNE1 NM_001238 amplification (CN 24); EZH2 NM_004456 deletion; TP53 NM_000546 c.700T>A p.Y234N	BROCA‐GO v1	yes	yes	5.6	yes	LOW
	931	IV	50‐54	YES	60	9	1) Carboplatin and Paclitaxel/ Gemcitabine (PR) 2) Carboplatin and Cediranib (SD) 3) Caelyx (PR) 4) Paclitaxel (1 month) (PD) 5) Carboplatin (PR) 6) Docetaxel (PD) 7) Carboplatin (1 month) (PD) 8) Cisplatin (SD) 9) Bevacizumab (PD)	None	BRCA2; c.1299_1331del, BRCA2; c.1300_1332del, BRCA2; c.1313dupA, TP53; c.743G>A p.R248Q	BROCA‐HR v8	yes	yes	42.9	yes	LOW
OCS	105	IIIC	45‐49	YES	37	1	1) Carboplatin and Paclitaxel (PD) 2) RTx	1) Carboplatin and Caelyx (PR) 2) Olaparib (maintenance) 3) Carboplatin and Paclitaxel (PD) 4) Adavosertib (PD)	RB1 NM_000321 c.1654C>T p.R552X TP53 NM_000546 c.725G>C p.C242S	BROCA‐HR v8	minimal	minimal	46.9	minimal	MED
	177	IIIC	60‐64	YES	16	1	1 cycle Carboplatin and Paclitaxel (PD)	1) Carboplatin and Paclitaxel (CR)	TP53 NM_000546 c.742C>T p.R248W TP53 NM_000546 c.818G>A p.R273H germline vus POLD1 NM_002691 c.2293G>A p.V765M	BROCA‐HR v8	yes	yes	0.2	no	LOW
	233	IIIA	50‐54	NO	10	0	None	1) Carboplatin and Paclitaxel (PD)	no mutations detected	BROCA‐HR v8	yes	yes	0.7	yes	LOW

*Note*: Patient clinical history, PDX molecular characteristics, and summary of PDX analysis from this study. None of the patients had received prior ADC therapy.

Abbreviations: Dx, diagnosis; PDX, patient‐derived xenograft; PD, progressive disease; Pt, Patient; PR, partial response; RTx, Radiotherapy; SD, stable disease; TTD, time to death.

**TABLE 2:** Uterine serous carcinoma (USC) and ovarian clear cell adenocarcinoma (OCCA) patient‐derived xenograft (PDX) treatment cohort summary.

Tumour type	PDX	Stage at Dx	Age at Dx	Patient treated prior to PDX	Patient TTD from diagnosis (months)	No. of prior systemic therapies	Pre‐treatment	Treatment in Pt subsequent to obtaining PDX	BROCA Molecular characterisation	BROCA panel version	paclitaxel response	eribulin response	% total FRA+	FZEC response	% total MSLN+	MORAb‐109 response	ABCB1 RT‐qPCR
USC	33	IIIC	45‐49 (USC) 40‐44(BC)	YES	44	2	1) RTx^*^ 2) Docetaxel, Cyclophosphamide (CR)^*^ 3) Tamoxifen (maintenance) ^*^ 1) Carboplatin and Paclitaxel (PD) 2) RTx 3) Caelyx (SD)	1) Durvalumab (PD)	TP53 c.844C>T, p.R282W TP53 c.451C>G, p.P151A PIK3CA c.3019G>C, p.G1007R XRCC2 c.731_732delAA; WRN c.1163delA;	BROCA‐HR v8	SD then PD	SD then PD	52.1	yes	38.1	yes	LOW
	69	IV	40‐44	YES	22	2	1) Carboplatin and Paclitaxel (MR) 2) Paclitaxel, Trastuzumab, Pertuzumab (PD)	1) T‐DM1 (PD) 2) Carboplatin and Gemcitabine (PD)	CCNE1 NM_001238 amplification (CN 24); CDK12 NM_016507 amplification (CN 9); ERBB2 NM_004448 amplification (CN 127); TP53 NM_000546 c.713G>T p.C238F; Single‐allele inactivation of BRCA1 and RPA2 due to genomic rearrangement	BROCA‐GO v1	no	no	4.1	no	7.7	no	HIGH
	116	II	60‐64	YES	31	1	1) RTx 2) Carboplatin and Paclitaxel (SD)	1) Lenvatinib (maintenance) 2) Letrozole (PD)	ARID1A NM_006015 c.1820C>G p.S607X; CCNE1 NM_001238 amplification (CN9); FBXW7 NM_001349798 c.1394G>A p.R465H; TP53 NM_000546 c.730G>A p.G244S	BROCA‐GO v1	no	no	53.9	no	2	no	HIGH
	155	IV	70‐74	NO	16	0	None	1) Carboplatin and Paclitaxel (PD) 2) Paclitaxel (SD) 3) Olaparib (PD)	Germline PALB2 NM_024675 c.2257C>T p.R753X, wildtype allele retained; TP53 NM_000546 c.818G>A p.R273H	BROCA‐GO v1	yes	yes	29.9	yes	3.4	no	LOW
	256	II	55‐59	YES	21	1	1) Carboplatin and Paclitaxel, PORTEC‐style (SD)	1) Trastuzumab. Pertuzumab, Paclitaxel (PR) 2) Carboplatin and Gemcitabine (PD) 3) T‐DM1 (PD)	ERBB2 NM_004448 amplification (CN 13); PIK3CA NM_006218 c.1636C>G p.Q546E; TP53 NM_000546 c.817C>T p.R273C; TP53 NM_000546 c.437G>A p.W146X; WNT7A—MYC rearrangement	BROCA‐GO v1	yes	yes	10.5	yes	58.9	yes	LOW
	410	II	70‐74 (USC) 60‐64 (CRC) 45‐50 (BC)	NO	11	0	1) Doxorubicin and Cyclophosphamide (CR)^*^ 1) 5FU/Capecitabine (PR^)^ ^**^ 2) FOLFOX and Bevacizumab (CR)^**^	1) Carboplatin and Paclitaxel (PD)	KRAS NM_004985 c.38G>A p.G13D; PIK3CA NM_006218 c.1624G>A p.E542K; TP53 NM_000546 c.775G>T p.D259Y	BROCA‐GO v1	yes	yes	32.8	yes	1.3	yes	LOW
OCCA	108	IV	40‐44	YES	23	2	1) Carboplatin, Paclitaxel and Bevacizumab (SD?) 2) Ipilimumab and Nivolumab (PR)	1) continued Ipi/Nivo (PR) 2) RTx (PD)	PIK3CA NM_006218 c.334A>T p.I112F	BROCA‐GO v1	SD then PD	yes	24.7	yes	22.6	yes	LOW
	279	IIIC	55‐59	YES	13	1	1) Carboplatin and Paclitaxel (CR)	1) Carboplatin, Paclitaxel and Bevacizumab (CR) 2) Cisplatin, Gemcitabine and Bevacizumab (PD)	SMARCA4 NM_001128844 c.3766C>T p.Q1256X; TP53 NM_000546 c.797G>A p.G266E; PIK3CA NM_006218 c.1624G>A p.E542K; WRN NM_000553 c.1676C>T p.S559L; ATM NM_000051 c.6452+138_6808‐70delinsAAGTCCTCAATGAATGGTAGTTGCTGCTTTC	BROCA‐HR v8	no	no	32.8	no	60.7	no	HIGH
	366	IV	70‐75	YES	10	1	1) Carboplatin, Paclitaxel and Bevacizumab (SD)	None	ARID1A NM_006015 c.546_555del p.A182fs; ATR NM_001184 c.7219C>A p.R2407S; FANCM NM_020937 c.359T>C p.I120T	BROCA‐GO v1	SD then PD	yes	51.1	yes	1.3	minimal	LOW

*Note*: Patient clinical history, PDX molecular characteristics, and summary of PDX analysis from this study.

Two of the USC models were derived from chemo‐naïve tumour samples (#155 and #410) with the remainder being derived from patients who had received treatment including carboplatin/paclitaxel, prior to the generation of the PDX. None of the patients had received prior ADC therapy. Two USC PDX models were HER2 positive, #69 and #256, in agreement with the patient tumour (Figure S2A), and for PDX #69 the patient had received chemotherapy combined with trastuzumab/pertuzumab treatment, prior to generation of the PDX model.

Abbreviations: CR, complete response; Dx, diagnosis; PDX, patient‐derived xenograft; PD, progressive disease; Pt, Patient; PR, partial response; RTx, Radiotherapy; SD, stable disease; TTD, time to death.

* breast cancer (BC) treatment

^**^colorectal cancer (CRC) treatment.

Whole genome sequencing of three of the drug resistant PDX identified a β‐tubulin mutation in PDX #69 (*TUBB2B* ENST00000259818.8, c.743C>T, p.Ala248Val, 0.15 adjusted allele frequency) that may contribute to AMA resistance (data not shown).^12^

All 15 aggressive GC PDX models showed impressive responses to FZEC and MORAb‐109 if positive for FRA and/or MSLN expression, respectively, and negative for high *ABCB1* expression. Given the paucity of therapeutic options available to patients with advanced GC, our data highlight the urgent need for inclusion of GC in eribulin‐ADC trials early in the GC journey; and exploration of high *ABCB1* expression as a potential exclusion criterion in clinical trials of ABCB1/P‐gp substrates.

## AUTHOR CONTRIBUTIONS

Ksenija Nesic—extracted RNA, performed qPCRs, analysed data, drafted and edited the manuscript. Katherine Rybinski—performed FRA and MSLN IHC and analysis using HALO. Gayanie Ratnayake—histological review. Ratana Lim—curation of patient data, edited the manuscript. Marc Radke–BROCA assay data analysis. Chloe Neagle—extracted RNA, performed qPCRs, analysed data. Gwo‐Yaw Ho and Matthew J. Wakefield—study design and initiation, edited the manuscript. Elizabeth M. Swisher—data analysis, edited the manuscript. Holly E. Barker—study design, IF analysis, edited the manuscript. Keiji Furuuchi—study design, IHC analysis using HALO, and edited manuscript. Clare L. Scott—study design and initiation, interpreted patient data, drafted and edited the manuscript. Cassandra J. Vandenberg—study design and initiation, developed and analysed preclinical models, led PDX treatment studies, analysed data, drafted and edited the manuscript. All authors read and approved the final manuscript.

## CONFLICT OF INTEREST STATEMENT

K. Nesic, R. Lim, C. Neagle, M. J. Wakefield, H.E. Barker, C.L. Scott and C.J. Vandenberg report research support (paid to institution) for this work from Eisai Inc; and outside the submitted work from AstraZeneca Pty Ltd and Boehringer Ingelheim, and non‐financial support from Clovis Oncology and Sierra Oncology. C.L. Scott reports unpaid advisory boards: AstraZeneca Pty Ltd, Clovis Oncology, Roche, Eisai, Sierra Oncology, Takeda, MSD. KR and KF were employees of Eisai Inc.

## FUNDING INFORMATION

This work was supported by funding from Eisai Inc. and grants from the Stafford Fox Medical Research Foundation (KN, RL, CN, MJW, HEB, CLS, CJV) and the National Health and Medical Research Council (NHMRC Australia; Investigator grant 2009783 (CLS)).

## ETHICS STATEMENT

Deidentified patient samples and corresponding clinical information were obtained from patients enrolled in the WEHI‐Stafford Fox Rare Cancer Program (SFRCP); all patients provided informed written consent. Ethics approval for the WEHI‐SFRCP was obtained from the Royal Melbourne Hospital Human Research Ethics Committee (Project number 2015.300) and governance review by the WEHI HREC (Project number G16/02). In addition, deidentified HGSOC samples were also obtained from patients consented to the Australian Ovarian Cancer Study (AOCS) at the Royal Women's Hospital (RWH HREC projects 01/56 and 10/57; WEHI HREC project 10/05).

## Supporting information



Supporting Information

Supporting Information

## Data Availability

The datasets generated during the current study are available from the corresponding author on reasonable request.

## References

[1] Cortes J , O'Shaughnessy J , Loesch D , et al. Eribulin monotherapy versus treatment of physician's choice in patients with metastatic breast cancer (EMBRACE): a phase 3 open‐label randomised study. Lancet. 2011;377(9769):914‐923. doi:10.1016/S0140-6736(11)60070-6 21376385

[2] Ho GY , Kyran EL , Bedo J , et al. Epithelial‐to‐Mesenchymal transition supports ovarian carcinosarcoma tumorigenesis and confers sensitivity to microtubule targeting with eribulin. Cancer Res. 2022;82(23):4457‐4473. doi:10.1158/0008-5472.CAN-21-4012 36206301 PMC9716257

[3] Ho GY , Vandenberg CJ , Lim R , et al. The microtubule inhibitor eribulin demonstrates efficacy in platinum‐resistant and refractory high‐grade serous ovarian cancer patient‐derived xenograft models. Ther Adv Med Oncol. 2023;15:17588359231208674. doi:10.1177/17588359231208674 38028140 PMC10666702

[4] Dumontet C , Reichert JM , Senter PD , Lambert JM , Beck A . Antibody‐drug conjugates come of age in oncology. Nat Rev Drug Discov. 2023;22(8):641‐661. doi:10.1038/s41573-023-00709-2 37308581

[5] Silverstein J , Karlan B , Herrington N , Konecny G . Antibody‐drug conjugates as targeted therapy for treating gynecologic cancers: update 2025. Curr Opin Obstet Gynecol. 2025;37(1):5‐15. doi:10.1097/GCO.0000000000001002 39480912 PMC11676621

[6] Cheng X , Li J , Tanaka K , et al. MORAb‐202, an antibody‐drug conjugate utilizing humanized anti‐human fralpha farletuzumab and the microtubule‐targeting agent eribulin, has potent antitumor activity. Mol Cancer Ther. 2018;17(12):2665‐2675. doi:10.1158/1535-7163.MCT-17-1215 30262588

[7] Albone EC , Cheng X .; Verdi A , Jacob S , et al. MORAb‐109: a site‐specific eribulin‐conjugated ADC targeting human mesothelin. Ann Oncol. 2020:S491‐S492.

[8] Shimizu T , Fujiwara Y , Yonemori K , et al. First‐in‐human phase 1 study of MORAb‐202, an antibody‐drug conjugate comprising farletuzumab linked to eribulin mesylate, in patients with folate receptor‐alpha‐positive advanced solid tumors. Clin Cancer Res. 2021;27(14):3905‐3915. doi:10.1158/1078-0432.CCR-20-4740 33926914

[9] Furuuchi K , Rybinski K , Fulmer J , et al. Antibody‐drug conjugate MORAb‐202 exhibits long‐lasting antitumor efficacy in TNBC PDx models. Cancer Sci. 2021;112(6):2467‐2480. doi:10.1111/cas.14898 33756060 PMC8177789

[10] Penson RT , Oliva E , Skates SJ , et al. Expression of multidrug resistance‐1 protein inversely correlates with paclitaxel response and survival in ovarian cancer patients: a study in serial samples. Gynecol Oncol. 2004;93(1):98‐106. doi:10.1016/j.ygyno.2003.11.053 15047220

[11] Robey RW , Pluchino KM , Hall MD , Fojo AT , Bates SE , Gottesman MM . Revisiting the role of ABC transporters in multidrug‐resistant cancer. Nat Rev Cancer. 2018;18(7):452‐464. doi:10.1038/s41568-018-0005-8 29643473 PMC6622180

[12] Yin S , Bhattacharya R , Cabral F . Human mutations that confer paclitaxel resistance. Mol Cancer Ther. 2010;9(2):327‐335. doi:10.1158/1535-7163.MCT-09-0674 20103599 PMC2820594

