# Supplementary material for: Farletuzumab ecteribulin and MORAb‐109, folate receptor alpha and mesothelin targeting antibody–drug conjugates, show activity in poor prognosis gynaecological cancer models
Source: Clin Transl Med. 2025 Mar 12;15(3):e70274. doi: 10.1002/ctm2.70274 (PMC11903193; doi:10.1002/ctm2.70274)
Supplement: Supplementary file 1 — Supporting Information [file CTM2-15-e70274-s002.docx]

**Nesic et al Supplementary Material**

**Extended Results**

Out of the 15 GC PDX models used for the FZEC studies we noted that there were two models that had a significant difference in survival between paclitaxel and eribulin, the OCCA models #108 and #366 (*P* = 0.0069 and *P* = 0.039, respectively), with eribulin showing the greater activity (Table S6). These models were low for *ABCB1* (Figure 2C), which led us to hypothesise that non-P-gp mechanisms of acquired resistance to paclitaxel do not have an equivalent impact on eribulin, or eribulin-ADC, responses. Eribulin treatment data for PDX models that were not selected for the FZEC and MORAb-109 studies, due to lack of antigen expression or lack of dual expression, reveal additional cases where eribulin shows greater benefit than paclitaxel (Figure S3, Tables S7 and S8). Interestingly, for two of these PDX models the patient had not received any prior paclitaxel treatment and yet the PDX was refractory (#333) or resistant (#295, short lived response) to paclitaxel. The only prior treatment for PDX #333 was radiotherapy, suggesting the possibility of intrinsic paclitaxel, but not eribulin, resistance. For two additional PDX models in this group the patient had not received a full course of carboplatin/paclitaxel treatment due to peripheral neuropathy (PN). Therefore, this group of PDX models is characterised by paclitaxel resistance with limited prior paclitaxel exposure and indicates a patient cohort that might benefit from eribulin.

**Extended Discussion**

ADCs are an emerging and highly promising approach for precision cancer therapy, particularly for cancers with limited standard therapeutic options^1,2^. In this study, we have demonstrated the potential utility of the FRA-targeting ADC, FZEC, and to a lesser extent, the MSLN-targeting ADC, MORAb-109, across several epithelial GC types, including USC and OCCA, which have few treatment options and poor outcomes in the clinic.

In the HGSOC group, FRA was expressed in the majority of PDX models screened (79%) and in all PDX models treated. FZEC response was observed consistently in FRA-positive models. Eribulin responses were also observed in all of these cases, in line with previous data^8^. However, FZEC achieved equal or greater duration of response with a considerably lower molar equivalent of eribulin. This is significant for patients, as the lower molar eribulin dosing may reduce eribulin side effects such as PN. The percentage of total tumour observed to be FRA positive used in this study, with a >5% FRA cut-off denoted as positive, is consistent with the first-in-human phase 1 clinical trial of FZEC’s safety and tolerability (NCT03386942). In that trial, a partial response was achieved in a breast cancer case that was only 13% FRA positive^9^. The FRA antibody used in both this study and the FZEC phase 1 clinical trial was mAb 26B3.F2. Incidentally, this is not the companion diagnostic for the ADC mirvetuximab soravtansine (MIRV), which uses the FDA-approved VENTANA FOLR1 RxDx assay with a cut-off for positivity being >75% positive tumour cells. Due to differences in antibodies and staining protocols, we were not able to directly equate FRA expression levels with the cut-offs for high, low and negative expression that have been used in MIRV studies^10^.

In OCS, FZEC response was found to correlate with eribulin response, in 2/3 PDXs, except in one PDX, #177, where FRA was not expressed. Perhaps surprisingly, FZEC response was observed in OCS PDX #233, which has only very low FRA expression. Notably, this model was extremely sensitive to eribulin, so in such cases the threshold level of target antigen expression required may be lower, supporting bystander effect as playing a major role in drug efficacy^11^. Additionally, it has been reported that FRA-dependent FZEC responses are observed at lower dose (5 mg/kg) but at higher dose (12.5 mg/kg), as used in this current study, some of the FRA dependence is lost^12^.

For three PDX models from the GC cohort, USC PDXs, #33 and #410 and OCCA #108, one or both ADC outperformed paclitaxel and eribulin (Figure 2A and 2B). For the clinical case #410, following collection of the sample that generated the PDX model, the patient received carboplatin and paclitaxel with progressive disease (Table 2). This clinical response is in keeping with that observed in PDX #410 and provides an example of a case in which FZEC would have potentially provided a better outcome. OCCA case #366 received only one line of treatment (carboplatin/paclitaxel and bevacizumab), and only achieved stable disease (Table 2). PDX #366 (FRA positive, MSLN negative) was generated following these treatments and had a very poor response to paclitaxel, however it demonstrated significantly improved responses to eribulin and FZEC. Along with PDX #108, this finding is in keeping with recent preclinical PDX work suggesting that eribulin has potential as a therapeutic agent for OCCA^13^**. Furthermore, given that all three OCCA models had FRA expression, and 2/3 had MSLN expression (#366 was negative, Figure S2B), the use of eribulin ADCs may be even more effective than standard eribulin (as observed for PDX #108), except in cases with ABCB1/P-gp overexpression (such as #279) (Figure 2C-F). In these cases, most P-gp substrate therapies, including eribulin, eribulin ADCs and other ADCs with MMAE and mertansine (DM1)-related payloads, are likely to be ineffective.**

Overall, deep and durable responses to FZEC were seen in 75% (9 out of 12) of FRA positive PDX models, including USC and OCCA. It is quite remarkable to achieve this outcome with a single agent therapy given at low dose in such aggressive GC PDX models. MSLN expression was not observed in all GC PDX models we treated with MORAb-109. Notably, for those models that were MSLN positive, response rates were 67% for USC (2 out of 3 PDX) and 50% for OCCA (1 out of 2 PDX).

Our data supports that eribulin can be an active chemotherapeutic in GC, as in breast cancer, particularly if used in the first line setting. Hence, it is important to assess the efficacy of eribulin-ADC-delivery earlier in the patient journey in the clinic. High expression of *ABCB1*, including by transcriptional fusions, has been reported in relapsed HGSOC and breast cancer and correlates with prior treatment with P-gp substrates, in particular paclitaxel^14,15^. Therefore, clinical trials evaluating eribulin or FZEC should be conducted for GC at first relapse (following only one line of paclitaxel) or in treatment exposure studies during first-line therapy. As regards to drug resistance due to P-gp, currently, there is no assay or accepted expression cut-off, validated for identifying clinically relevant levels of *ABCB1* expression, perhaps due to differences in expression between cancer types^15,16^. In our PDX models, even low expression of *ABCB1* appeared to lead to eribulin and eribulin-ADC resistance. A clinical test for *ABCB1* could prevent the use of ineffective treatments and will be an important step in the future development of ADCs (with P-gp substrate payload) as a drug class, as could the development of combination of ADCs with P-gp inhibitor therapy.

ADCs with non-overlapping toxicities, when used in combination with chemotherapy or other targeted therapeutics, have the potential to expand therapeutic options for treating poor prognosis GC. Encouragingly, MIRV in combination with carboplatin and bevacizumab, has a tolerable safety profile^17,18^**. Eribulin has been shown to have beneficial properties in addition to microtubule inhibition, including vascular remodelling, enhancing infiltration of lymphocytes, and inhibition of epithelial-to-mesenchymal transition^19-21^. Whether FZEC possesses these properties is yet to be determined^9^, but could provide a point of difference compared with other FRA-targeting ADCs in development for GC.**

**Methods**

**Study approvals**

Deidentified patient samples and corresponding clinical information were obtained from patients enrolled in the WEHI-Stafford Fox Rare Cancer Program (SFRCP); all patients provided informed written consent. Ethics approval for the WEHI-SFRCP was obtained from the Royal Melbourne Hospital Human Research Ethics Committee (Project number 2015.300) and governance review by the WEHI HREC (Project number G16/02). In addition, deidentified HGSOC samples were also obtained from patients consented to the Australian Ovarian Cancer Study (AOCS) at the Royal Women’s Hospital (RWH HREC projects 01/56 and 10/57; WEHI HREC project 10/05).

Procedures involving animals were approved by the WEHI animal ethics committee (Project number 2019.024) and performed in accordance with the Australian Code for the Care and Use of Animals for Scientific Purposes 8th Edition, 2013 (updated 2021).

**Generation and treatment of PDX models**

HGSOC PDX models #206, #931 and #111 have been published previously^8,22^. OCS PDX models #105 and #177 have been published previously^20^. The WEHI-SFRCP cohort of rare GC PDX models were generated by subcutaneous transplantation of fresh biopsy or surgical tumour fragments (<2 mm in each dimension) into NOD.Cg-Prkdc^scid^ Il2rg^tm1Wjl^/SzJ (NSG) mice. Mice were assessed weekly for the growth of PDX tumours and these were harvested once they were >700 mm^3^, but before they reached the maximum ethically approved tumour volume of 1000 mm^3^. New PDX models were verified histologically and by BROCA panel or other sequencing comparison with the patient tumour. STR profiling was performed as a cost-effective method for future ID confirmation.

PDX models were serially passaged by surgical implantation of tumour fragment subcutaneously into NSG mice, for up to 10 passages. For OCS PDX models, the composition (% carcinoma vs % sarcoma) of tumours is determined by the composition of the baseline patient sample and subsequently by the tumour fragment composition, which can be variable. Tumours were measured twice weekly with digital callipers and automatically recorded using StudyLog Desktop software (StudyLog Systems Inc, CA, USA). At tumour volume 180-300 mm^3^ mice were randomised to treatment: eribulin (Eisai Inc.) 1 mg/kg was administered intraperitoneally (10 µL/g), 3 times weekly for three weeks; FZEC (Eisai Inc.) 12.5 mg/kg was administered by intravenous injection (5 µL/g) once, or repeat dosing as described in figure legends; MORAb-109 (Eisai Inc.) 25 mg/kg was administered by intravenous injection (5 µL/g) fortnightly for three doses; cisplatin (Pfizer) 4 mg/kg was given by intraperitoneal injection (20 µL/g) on days 1, 8 and 18; paclitaxel (Sandoz) 25 mg/kg was delivered twice weekly for three weeks by intraperitoneal injection (20 µL/g). All drugs were prepared at the required dosing concentration in DPBS (Gibco). The paclitaxel formulation also contained 100 IU/mL hyaluronidase (Hyalase, Sanofi Aventis) to improve absorption. Experimental endpoints were tumour volume >700 mm^3^ (prior to ethical endpoint for tumour volume of 1000 mm^3^) or 120 days following randomisation (for some models this was extended to 150 days).

SurvivalVolume was used to graph tumour volume and Kaplan-Meier curves and for statistical analysis^23^.

**Immunohistochemistry**

PDX tumour samples were fixed in 10% formalin for 24-48h before embedding in paraffin. Sections were stained with haematoxylin and eosin (H&E) and automated immunohistochemistry (IHC) performed using a Dako Omnis (Agilent, CA, USA) and the following antibodies: anti-pan-cytokeratin clone AE1/3 (M3515, Dako), anti-PAX8 polyclonal (10336-1-AP, Proteintech), anti-p53 clone DO-7 (M700101, Dako), anti-vimentin clone D21H3 (5741, Cell Signaling Technology), anti-HER2 clone SP3 (ab16662, Abcam), anti-folate receptor alpha (FRA) clone 26B3^11^, and anti-mesothelin (MSLN) clone 15C11 (AC13175B, Biocare).

Stained slides were scanned with a Pannoramic Scan II (3DHISTECH Ltd) and images uploaded to CaseCenter (3DHISTECH LTD.). Snapshots from CaseCenter were prepared for publication using FIJI^24^. For IHC quantification HALO^Ò^ image analysis software was used (Indica Lab, RRID:SCR_018350). First the tumour region was defined, with necrotic areas excluded, then tumour and stroma areas were defined by the tissue classifier module, followed by quantification of the percentage of FRA or MSLN positive cells in the tumour classified area was determined by the area quantification module.

**DNA sequencing**

BROCA next-generation sequencing assays (University of Washington, Seattle, WA, USA) were used to molecularly characterise PDX models. BROCA-HR v8 was used for PDX #206, #931, #105, #177, #233, #33 and #279; and BROCA-GO v1 was used for #111, #69, #116, #155, #256, #410, #108, and #366 (https://testguide.labmed.uw.edu/view/BROCA).

***ABCB1* qRT-PCRs**

*ABCB1* qRT-PCRs were performed as previously described^22^. Briefly, vehicle and untreated PDX samples were selected at random for assessment of baseline *ABCB1* gene expression levels. RNA was extracted from tissue lysates using the Direct-zol RNA MiniPrep (Zymo Research, Cat# R2050) according to the manufacturer’s protocol. RNA was quantitated using the NanoDrop 2000 UV-Vis Spectrophotometer (Thermo Fisher Scientific, Cat# ND-2000). SuperScript III Reverse Transcriptase (Invitrogen, Cat# 18080085) was used to convert RNA to cDNA according to the manufacturer’s protocol. A volume of 2 µL cDNA diluted to 5 ng/µL was added to 5 µL SYBR Green PCR Master Mix (Thermo Fisher Scientific), 2.5 µL molecular-grade H_2_O and 0.5 µL of the relevant 2 µM primer mix (Methods Table 1). *ABCB1* primers used were as previously published^15^.

Plates were incubated on the ABI 7900 (Applied Biosystems) or the ViiA 7 Real-Time PCR System (Thermo Fisher Scientific) at 95°C for 10 minutes, 40 cycles of 95°C for 15 seconds and 60°C for 1 minute*, followed by 95°C for 15 seconds, 60°C for 15 seconds and 95°C for 15 seconds* (*Data recorded at indicated steps). Resulting CT values were normalised to the averaged value housekeeper control primers in Excel (Microsoft). Fold change values were calculated for each sample. These values were then graphed in PRISM7 (GraphPad), and the standard deviation calculated.

***Methods Table 1.*** *HPRT, B-ACTIN, SDHA and GAPDH were used as housekeeper control primers. ABCB1 primers are previously published^15^****.***

**Table S1 Gynaecological cancer PDX cohort summary**

| **Tumour type** | **No. of PDX models** | **Derived from chemo-naïve sample  n (%)** | **No. of prior systemic therapies in patient** | **total FRA positive >5%  n (%)** | **total MSLN positive >5% n (%)** |
| --- | --- | --- | --- | --- | --- |
| HGSOC | 14 | 7 (50%) | 0 - 9 | 11 (79%) | ND |
| OCS | 3 | 1 (33%) | 0 - 1 | 1 (33%) | 0 (0%) |
| OCCA | 3 | 0 (0%) | 1 - 2 | 3 (100%) | 2 (67%) |
| Large cell NET ovary | 1 | 0 (0%) | 3 | 0 (0%) | 0 (0%) |
| USC | 8 | 2 (25%) | 0 - 2 | 6 (75%) | 3 (38%) |
| UCS | 5 | 1 (20%) | 0 - 3 | 2 (40%) | 0 (0%) |
| uLMS | 2 | 1 (50%) | 0 - 3 | 0 (0%) | 0 (0%) |
| Uterine Adenosarcoma | 1 | 1 (100%) | 0 | 0 (0%) | 0 (0%) |
| Cervical Adenocarcinoma | 2 | 0 (0%) | 1 - 2 | 0 (0%) | 1 (50%) |
| Cervical SCC | 1 | 0 (0%) | 3 | 1 (100%) | 0 (0%) |
| Vulval SCC | 1 | 1 (100%) | 0 | 0 (0%) | 0 (0%) |

Cohort of PDX models screened for FRA and MSLN expression.

HGSOC, high-grade serous ovarian carcinoma; OCS, ovarian carcinosarcoma; OCCA, ovarian clear cell adenocarcinoma; NET, neuroendocrine tumour; USC, uterine serous carcinoma; UCS; uterine carcinosarcoma; uLMS, uterine leiomyosarcoma; SCC, squamous cell carcinoma; ND, not determined.

Clinical details are provided in Tables 1 and 2 for PDX models selected for treatment.

**Table S2 FRA scoring of HGSOC PDX models**

|  | **HGSOC PDX model** | **Prior systemic therapies in patient** | **% total FRA positive** |
| --- | --- | --- | --- |
| **Chemo-naïve** | 11 | 0 | **13.8** |
|  | 13 | 0 | 2.4 |
|  | 29 | 0 | 0.2 |
|  | 62 | 0 | **37.9** |
|  | 183 | 0 | **24.9** |
|  | 197 | 0 | **45.7** |
|  | 201 | 0 | **44.2** |
| **Post-treatment** | 32 | 5 | **55.7** |
|  | 49 | 4 | **51.6** |
|  | 111 | 4 | **5.6** |
|  | 153 | 4 | **6.5** |
|  | 169 | 2 | 0.4 |
|  | 206 | 1 | **32.0** |
|  | 931 | 9 | **42.9** |

FRA IHC was quantified using HALO software. First the total tumour region was defined then the percentage of the tumour region that is FRA positive quantified. We selected 5% total tumour as our cut-off for positive staining as a previous analysis in triple negative breast cancer PDX models showed 4% total tumour staining was sufficient for significant growth inhibition with FZEC treatment^11^. No significant difference in the proportion of FRA positivity was observed between chemo-naïve and post-treatment groups. Blue shading indicates the PDX models that were selected for FZEC treatment studies. Bold text is used for values >5%.

**Table S3 FZEC treatment statistics for HGSOC and OCS PDX models**

| **PDX model** | **Tumour type** | **Treatment** | **n** | **Time to PD** | **CR  n (%)** | **Median TTH** | **p value vs vehicle** | **p value vs cisplatin** | **p value vs paclitaxel** | **p value vs eribulin** | **p value vs FZEC single dose** |
| --- | --- | --- | --- | --- | --- | --- | --- | --- | --- | --- | --- |
| #206 | HGSOC | vehicle | 16 | 8 | 0 (0) | 50 |  |  |  |  |  |
|  |  | cisplatin | 6 | >95 | 2 (33) | >120 | 0.0017 |  |  |  |  |
|  |  | paclitaxel | 6 | >120 | 6 (100) | >120 | 0.0014 | 1 |  |  |  |
|  |  | eribulin | 6 | >120 | 4 (67) | >120 | 0.0011 | 1 | 1 |  |  |
|  |  | FZEC single dose | 6 | 106 | 6 (100) | >120 | 0.0020 | 0.56 | 0.36 | 0.48 |  |
| #931 | HGSOC | vehicle | 15 | 8 | 0 (0) | 25 |  |  |  |  |  |
|  |  | cisplatin | 9 | 8 | 0 (0) | 43 | 0.0069 |  |  |  |  |
|  |  | paclitaxel | 8 | 106 | 8 (100) | >120 | 0.0000 | 0.0000 |  |  |  |
|  |  | eribulin | 8 | 99 | 5 (63) | >120 | 0.0000 | 0.0000 | 0.26 |  |  |
|  |  | FZEC single dose | 7 | 109 | 6 (86) | >120 | 0.0001 | 0.0001 | 0.24 | 0.97 |  |
| #111 | HGSOC | vehicle | 14 | 8 | 0 (0) | 43 |  |  |  |  |  |
|  |  | cisplatin | 9 | 15 | 0 (0) | 64 | 0.0020 |  |  |  |  |
|  |  | paclitaxel | 10 | 85 | 7 (70) | >120 | 0.0000 | 0.0000 |  |  |  |
|  |  | eribulin | 6 | 99 | 5 (83) | >120 | 0.0001 | 0.0010 | 0.67 |  |  |
|  |  | FZEC single dose | 7 | 85 | 6 (86) | >120 | 0.0000 | 0.0001 | 0.37 | 0.50 |  |
|  |  | FZEC d1,8,15 | 5 | >120 | 5 (100) | >120 | 0.0004 | 0.0009 | 0.059 | **0.034** | 0.052 |
| #105 | OCS | vehicle | 17 | 8 | 0 (0) | 18 |  |  |  |  |  |
|  |  | cisplatin | 11 | 8 | 0 (0) | 46 | 0.0018 |  |  |  |  |
|  |  | paclitaxel | 8 | 8 | 0 (0) | 46 | 0.11 | 0.39 |  |  |  |
|  |  | eribulin | 9 | 8 | 0 (0) | 39 | 0.032 | 0.48 | 0.57 |  |  |
|  |  | FZEC single dose | 8 | 8 | 0 (0) | 53 | 0.0058 | 0.58 | 0.43 | 0.79 |  |
| #177 | OCS | vehicle | 19 | 8 | 0 (0) | 15 |  |  |  |  |  |
|  |  | cisplatin | 7 | 8 | 0 (0) | 29 | 0.010 |  |  |  |  |
|  |  | paclitaxel | 7 | 81 | 2 (29) | 116 | 0.0000 | 0.0002 |  |  |  |
|  |  | eribulin | 7 | >120 | 5 (71) | >120 | 0.0000 | 0.0006 | 0.39 |  |  |
|  |  | FZEC single dose | 7 | 8 | 0 (0) | 25 | 0.016 | 0.40 | 0.0002 | **0.0002** |  |
| #233 | OCS | vehicle | 20 | 8 | 0 (0) | 22 |  |  |  |  |  |
|  |  | cisplatin | 8 | 8 | 0 (0) | 29 | 0.029 |  |  |  |  |
|  |  | paclitaxel | 8 | >120 | 8 (100) | >120 | 0.0000 | 0.0000 |  |  |  |
|  |  | eribulin | 8 | >120 | 7 (88) | >120 | 0.0000 | 0.0000 | 1 |  |  |
|  |  | FZEC single dose | 7 | >120 | 6 (86) | >120 | 0.0000 | 0.0001 | 0.32 | 0.32 |  |

Statistics for the HGSOC and OCS PDX treatment data presented in Figures 1B and 1D.

PD, progressive disease (as defined by Topp et al 2014); CR, complete response (<50mm^3^ for 3 consecutive weeks); TTH, time to harvest (days). P values for Kaplan-Meier survival curves were determined using the Log-Rank test. P values in bold indicate a significant difference between eribulin and FZEC (P value <0.05).

**Table S4 FRA and MSLN scoring of OCS PDX models**

|  | **OCS PDX model** | **Prior systemic therapies in patient** | **% total**  **FRA positive** | **% total MSLN positive** |
| --- | --- | --- | --- | --- |
| **Chemo-naïve** | 233 | 0 | 0.7 | 0 |
| **Post-treatment** | 105 | 1 | **46.9** | 4.7 |
|  | 177 | 1 | 0.2 | 0.1 |

FRA and MSLN IHC on OCS PDX models were analysed using HALO software. First the total tumour region was defined then the percentage of the tumour region that is FRA positive or MSLN positive was quantified. Bold text is used for values >5%. Blue shading indicates the PDX models that were selected for FZEC treatment studies.

**Table S5 FRA and MSLN scoring of rare GC PDX models**

| **Tumour type** | **PDX model** | **Prior systemic therapies in patient** | **% total FRA**  **positive** | **% total MSLN positive** |
| --- | --- | --- | --- | --- |
| OCCA | 108 | 2 | **24.7** | **22.6** |
|  | 279 | 1 | **32.8** | **60.7** |
|  | 366 | 1 | **51.1** | 1.3 |
| Large cell NET ovary | 143 | 3 | 0.5 | -0.4 |
| USC | 33 | 2 | **52.1** | **38.1** |
|  | 63 | 1 | **30.7** | -0.8 |
|  | 69 | 2 | 4.1 | **7.7** |
|  | 116 | 1 | **53.9** | 2.0 |
|  | 155 | 0 | **29.9** | 3.4 |
|  | 178 | 1 | 0.4 | 0.0 |
|  | 256 | 1 | **10.5** | **58.9** |
|  | 410 | 0 | **32.8** | 1.3 |
| UCS | 68 | 3 | 0.1 | -0.2 |
|  | 73 | 2 | **41.3** | -0.4 |
|  | 131 | 2 | 0.1 | 0.4 |
|  | 342 | 0 | **7.2** | 0.8 |
|  | 343 | 1 | 2.3 | 0.0 |
| uLMS | 122 | 3 | 0.5 | 0.0 |
|  | 227 | 0 | 1.4 | 0.5 |
| Uterine Adenosarcoma | 270 | 0 | 0.1 | 0.0 |
| Cervical Adenocarcinoma | 214 | 2 | 0.1 | **33.1** |
|  | 295 | 1 | 1.0 | 0.0 |
| Cervical SCC | 271 | 3 | **5.2** | -0.1 |
| Vulval SCC | 219 | 0 | 1.1 | 1.1 |

FRA and MSLN IHC on rare GC PDX models were analysed using HALO software. First the total tumour region was defined then the percentage of the tumour region that is FRA positive or MSLN positive was quantified. Bold text indicates values >5%.

Blue shading indicates the PDX models that were selected for FZEC and MORAb-109 treatment studies; PDX models with dual positivity were favoured.

OCCA, ovarian clear cell adenocarcinoma; NET, neuroendocrine tumour; USC, uterine serous carcinoma; UCS, uterine carcinosarcoma; uLMS, uterine leiomyosarcoma; SCC, squamous cell carcinoma.

**Table S6 FZEC and MORAb-109 treatment statistics for USC and OCCA PDX models**

| **PDX model** | **Tumour type** | **Treatment** | **n** | **Time to PD** | **CR  n (%)** | **Median TTH** | **p value vs vehicle** | **p value vs cisplatin** | **p value vs paclitaxel** | **p value vs eribulin** | **p value vs FZEC d1,15,29** |
| --- | --- | --- | --- | --- | --- | --- | --- | --- | --- | --- | --- |
| #33 | USC | vehicle | 9 | 8 | 0 (0) | 15 |  |  |  |  |  |
|  |  | cisplatin | 8 | 39 | 0 (0) | 60 | 0.0003 |  |  |  |  |
|  |  | paclitaxel | 8 | 53 | 0 (0) | 60 | 0.0006 | 0.72 |  |  |  |
|  |  | eribulin | 8 | 50 | 0 (0) | 67 | 0.0000 | 0.067 | 0.65 |  |  |
|  |  | FZEC d1,15,29 | 7 | >120 | 6 (86) | >120 | 0.0003 | 0.0032 | 0.0078 | **0.0029** |  |
|  |  | MORAb-109 d1,15,29 | 7 | >120 | 5 (71) | >120 | 0.0001 | 0.0002 | 0.0012 | **0.0001** | 0.91 |
| #69 | USC | vehicle | 18 | 8 | 0 (0) | 39 |  |  |  |  |  |
|  |  | cisplatin | 8 | 8 | 0 (0) | 46 | 0.66 |  |  |  |  |
|  |  | paclitaxel | 8 | 8 | 0 (0) | 32 | 0.31 | 0.14 |  |  |  |
|  |  | eribulin | 9 | 8 | 0 (0) | 36 | 0.92 | 0.62 | 0.62 |  |  |
|  |  | FZEC d1,15,29 | 8 | 8 | 0 (0) | 43 | 0.92 | 0.48 | 0.33 | 0.63 |  |
|  |  | MORAb-109 d1,15,29 | 7 | 8 | 0 (0) | 39 | 0.73 | 0.29 | 0.38 | 0.99 | 0.93 |
| #116 | USC | vehicle | 16 | 8 | 0 (0) | 22 |  |  |  |  |  |
|  |  | cisplatin | 7 | 8 | 0 (0) | 32 | 0.029 |  |  |  |  |
|  |  | paclitaxel | 7 | 8 | 0 (0) | 32 | 0.019 | 0.63 |  |  |  |
|  |  | eribulin | 7 | 8 | 0 (0) | 25 | 0.34 | 0.64 | 0.43 |  |  |
|  |  | FZEC d1,15,29 | 6 | 8 | 0 (0) | 32 | 0.24 | 0.33 | 0.30 | 0.99 |  |
|  |  | MORAb-109 d1,15,29 | 6 | 8 | 0 (0) | 22 | 0.28 | 0.0064 | 0.0025 | 0.13 | 0.082 |
| #155 | USC | vehicle | 14 | 8 | 0 (0) | 36 |  |  |  |  |  |
|  |  | cisplatin | 8 | 8 | 0 (0) | 64 | 0.017 |  |  |  |  |
|  |  | paclitaxel | 7 | 64 | 4 (57) | >140 | 0.0000 | 0.0027 |  |  |  |
|  |  | eribulin | 8 | 85 | 2 (25) | 106 | 0.0000 | 0.0002 | 0.32 |  |  |
|  |  | FZEC d1,15,29 | 6 | >120 | 5 (83) | >140 | 0.0001 | 0.0004 | 0.10 | **0.0011** |  |
|  |  | MORAb-109 d1,15,29 | 6 | 8 | 0 (0) | 39 | 0.43 | 0.55 | 0.0041 | **0.0005** | 0.0005 |
| #256 | USC | vehicle | 13 | 8 | 0 (0) | 32 |  |  |  |  |  |
|  |  | cisplatin | 7 | 8 | 0 (0) | 64 | 0.34 |  |  |  |  |
|  |  | paclitaxel | 7 | 85 | 4 (57) | >120 | 0.0075 | 0.018 |  |  |  |
|  |  | eribulin | 6 | >120 | 6 (100) | >120 | 0.0013 | 0.0006 | 0.31 |  |  |
|  |  | FZEC d1,15,29 | 7 | >120 | 6 (86) | >120 | 0.0005 | 0.0004 | 0.21 | 0.85 |  |
|  |  | MORAb-109 d1,15,29 | 6 | >120 | 6 (100) | >120 | 0.0005 | 0.0004 | 0.081 | 0.27 | 0.35 |
| #410 | USC | vehicle | 16 | 8 | 0 (0) | 18 |  |  |  |  |  |
|  |  | cisplatin | 7 | 8 | 0 (0) | 22 | 0.81 |  |  |  |  |
|  |  | paclitaxel | 7 | 53 | 0 (0) | 81 | 0.0000 | 0.0001 |  |  |  |
|  |  | eribulin | 7 | 53 | 0 (0) | 71 | 0.0000 | 0.0001 | 0.38 |  |  |
|  |  | FZEC d1,15,29 | 6 | 113 | 4 (67) | >120 | 0.0001 | 0.0004 | 0.026 | **0.017** |  |
|  |  | MORAb-109 d1,15,29 | 6 | 57 | 0 (0) | 88 | 0.0001 | 0.0004 | 0.19 | 0.080 | 0.051 |
| #108 | OCCA | vehicle | 7 | 8 | 0 (0) | 18 |  |  |  |  |  |
|  |  | cisplatin | 7 | 8 | 0 (0) | 32 | 0.031 |  |  |  |  |
|  |  | paclitaxel | 7 | 43 | 0 (0) | 64 | 0.0002 | 0.0003 |  |  |  |
|  |  | eribulin | 7 | 67 | 4 (57) | 78 | 0.0002 | 0.0001 | 0.0069* |  |  |
|  |  | FZEC d1,15,29 | 7 | 116 | 7 (100) | 137 | 0.0002 | 0.0001 | 0.0002 | **0.0003** |  |
|  |  | MORAb-109 d1,15,29 | 7 | >140 | 7 (100) | >140 | 0.0002 | 0.0001 | 0.0002 | **0.0003** | 0.52 |
| #279 | OCCA | vehicle | 7 | 8 | 0 (0) | 18 |  |  |  |  |  |
|  |  | cisplatin | 7 | 8 | 0 (0) | 18 | 0.81 |  |  |  |  |
|  |  | paclitaxel | 7 | 8 | 0 (0) | 32 | 0.023 | 0.11 |  |  |  |
|  |  | eribulin | 7 | 8 | 0 (0) | 32 | 0.065 | 0.16 | 0.55 |  |  |
|  |  | FZEC d1,15,29 | 7 | 8 | 0 (0) | 29 | 0.60 | 0.99 | 0.046 | 0.20 |  |
|  |  | MORAb-109 d1,15,29 | 7 | 29 | 0 (0) | 22 | 0.18 | 0.18 | 0.58 | 0.90 | 0.25 |
| #366 | OCCA | vehicle | 11 | 8 | 0 (0) | 25 |  |  |  |  |  |
|  |  | cisplatin | 7 | 8 | 0 (0) | 32 | 0.029 |  |  |  |  |
|  |  | paclitaxel | 7 | 8 | 0 (0) | 50 | 0.0009 | 0.12 |  |  |  |
|  |  | eribulin | 7 | 71 | 1 (14) | 92 | 0.0001 | 0.0001 | 0.039* |  |  |
|  |  | FZEC d1,15,29 | 7 | 95 | 1 (14) | 120 | 0.0001 | 0.0001 | 0.048 | 0.43 |  |
|  |  | MORAb-109 d1,15,29 | 7 | 8 | 0 (0) | 43 | 0.0030 | 0.38 | 0.62 | **0.033** | 0.048 |

Statistics for the USC and OCCA PDX treatment data presented in Figure 2A and 2B.

PD, progressive disease (as defined by Topp et al 2014); CR, complete response (<50mm^3^ for 3 consecutive weeks); TTH, time to harvest (days). P values for Kaplan-Meier survival curves were determined using the Log-Rank test. P values in bold indicate a significant difference between eribulin and FZEC, or eribulin and MORAb-109 (P value <0.05). *Indicates a significant difference between eribulin and paclitaxel, which was observed in only 2/15 PDX models.

**Table S7 GC cohort not selected for PDX ADC treatment experiments**

| **PDX** | **Tumour type** | **Age at Dx** | **TTD from diagnosis (months)** | **No. of prior systemic therapies** | **Pre-treatment** | **Treatment in Pt subsequent to obtaining PDX** |
| --- | --- | --- | --- | --- | --- | --- |
| #343 | UCS | 75-79 | 22 | 1 | 1) RTx (pelvis) 2) Carboplatin and Paclitaxel x6 (DR for C5 and C6, SD) 3) RTx (pelvis), (PR) | nil |
| #465 | High grade endometrioid adenocarcinoma | 75-79 | 31 | 3 | 1) Carboplatin and Paclitaxel X4, then Carbo x2 2) Trastuzumab and Pertuzumab 3) Caelyx | nil |
| #333 | Vulval adenocarcinoma (arising from mammary cells) | 55-59 | 26 | 0 | 1) RTx | 1) Cisplatin 2) Trastuzumab, Pertuzumab, Paclitaxel 3) Trastuzumab Deruxtecan (TDX-d) |
| #214 | Adenocarcinoma of the Cervix | 35-39 | 24 | 2 | 1) Carboplatin, Paclitaxel, Bevacizumab x6 2) ChemoRT (Cisplatin x1, then RTx alone) 3) Brachytherapy | 1) Carboplatin and Paclitaxel |
| #295 | Adenocarcinoma of the Cervix | 40-44 | 203 | 1 | 1) ChemoRT (Cisplatin) 2) Brachytherapy | Tissue collection from first recurrencce since 2006 1) Carboplatin, Paclitaxel and Bevacizumab 2) Bevacizumab (maintenance) (with RTx to brain mets) 3) clinical trial AK104 (an anti-PD1 and anti-CTLA4 bispecific antibody) 4) Carboplatin and Paclitaxel x3 5) RTx to brain mets 6) Capecitabine |
| #271 | Cervical SCC | 35-39 | NA | 3 | 1) ChemoRT (Cisplatin) 2) Carboplatin and Paclitaxel X4 3) Carboplatin, Paclitaxel, Bevacizumab x6 | 1) Ipilimumab and Nivolumab x3 (CR) |

TTD, time to death; Pt, Patient; Dx, diagnosis; RTx, Radiotherapy; CR, complete response; PR, partial response; SD, stable disease; PD, progressive disease; DR, dose reduction; UCS, uterine carcinosarcoma; SCC, squamous cell carcinoma.

**Table S8 Eribulin treatment statistics for GC PDX models**

| **PDX model** |  | **Treatment** | **n** | **Time to PD** | **Median TTH** | **p value vs vehicle** | **p value vs cisplatin** | **p value vs paclitaxel** |
| --- | --- | --- | --- | --- | --- | --- | --- | --- |
| #343 | Uterine carcinosarcoma | vehicle | 11 | 8 | 32 |  |  |  |
|  |  | cisplatin | 7 | 50 | >120 | 0.0001 |  |  |
|  |  | paclitaxel | 7 | 25 | 85 | 0.0006 | 0.11 |  |
|  |  | eribulin | 7 | >120 | >120 | 0.0001 | 0.0075 | **0.0008** |
| #465 | High grade endometrioid adenocarcinoma | vehicle | 11 | 8 | 22 |  |  |  |
|  |  | cisplatin | 7 | 8 | 18 | 0.54 |  |  |
|  |  | paclitaxel | 7 | 8 | 25 | 0.15 | 0.74 |  |
|  |  | eribulin | 6 | 36 | 57 | 0.0001 | 0.050 | **0.0065** |
| #333 | Vulval adenocarcinoma (arising from Paget's disease) | vehicle | 6 | 8 | 11 |  |  |  |
|  |  | cisplatin | 4 | 8 | 18 | 0.020 |  |  |
|  |  | paclitaxel | 5 | 8 | 22 | 0.0063 | 0.34 |  |
|  |  | eribulin | 4 | 71 | 92 | 0.0038 | 0.0067 | **0.0060** |
| #214 | Cervical adenocarcinoma | vehicle | 8 | 8 | 29 |  |  |  |
|  |  | cisplatin | 8 | 25 | 60 | 0.0000 |  |  |
|  |  | paclitaxel | 7 | 60 | 81 | 0.0001 | 0.020 |  |
|  |  | eribulin | 7 | >120 | >120 | 0.0001 | 0.0006 | **0.020** |
| #295 | Cervical adenocarcinoma | vehicle | 7 | 11 | 67 |  |  |  |
|  |  | cisplatin | 6 | 50 | 88 | 0.017 |  |  |
|  |  | paclitaxel | 5 | 50 | 113 | 0.0020 | 0.066 |  |
|  |  | eribulin | 7 | 95 | >120 | 0.0014 | 0.013 | **0.0065** |
| #271 | Cervical squamous cell carcinoma | vehicle | 13 | 8 | 11 |  |  |  |
|  |  | cisplatin | 7 | 8 | 36 | 0.0006 |  |  |
|  |  | paclitaxel | 6 | 8 | 18 | 0.57 | 0.0008 |  |
|  |  | eribulin | 8 | 36 | 67 | 0.0007 | 0.040 | **0.0084** |

Statistics for the RGC PDX treatment data presented in Supplementary Figure 3

PD, progressive disease (as defined by Topp et al 2014); TTH, time to harvest (days); *P* values for Kaplan-Meier survival curves were determined using the Log-Rank test. *P* values in bold indicate a significant difference between paclitaxel and eribulin (*P* value <0.05).

**Figure S1 Prolonged complete response with repeated FZEC dosing.**

Mice bearing HGSOC PDX model #111 were treated with (A) eribulin (1 mg/kg, 3 times weekly for 3 weeks; black bar), or (B) FZEC single dose (12.5 mg/kg, IV; black arrow), or (C) FZEC weekly dosing for 3 weeks (12.5 mg/kg, IV; black arrows). Spaghetti plots representing the tumour volume for each mouse post randomisation. Mice that had a complete response (CR, tumour volume less than 50 mm^3^ for three consecutive weeks) are shown in red. All mice treated with three doses of FZEC had a CR and did not progress before the experimental endpoint of 120 days. (D) Change in body weight of mice was evaluated during treatment. The data shown corresponds to the PDX #111 treatment mice in Figure 1B. Mice were weighed prior to each treatment dose. Weekly dosing with FZEC (12.5 mg/kg) for 3 weeks did not cause weight loss. Mean ± SEM.

**Figure S2 Evaluation of FRA and MSLN expression in rare GC PDX models.**

Morphology and expression of GC markers (PanCK, Pax8, p53) in (A) USC PDX models and (B) OCCA PDX models were reviewed by a gynaecological pathologist and confirmed as consistent with the patients’ pathology reports. (A) All six USC PDX models showed mutation type expression of p53, and two *ERBB2* amplified PDX models, #69 and #256, had high HER2 expression as expected. FRA expression was evident in 5/6 USC PDX models and MSLN in 3/6. Scale bar represents 100 µm. (B) In 2/3 OCCA PDX models, #108 and #366, wildtype staining for p53 was seen. High FRA expression was apparent in all three OCCA models and high MSLN in 2/3. (C) Change in body weight of mice was evaluated during treatment. The data shown corresponds to the PDX #33 and #108 treatment mice in Figure 2. Mice were weighed prior to each treatment dose. Fortnightly dosing with FZEC (12.5 mg/kg) or MORAb-109 (25mg/kg) for 3 doses did not cause weight loss. Mean ± SEM.

**Figure S3 Eribulin activity independent of paclitaxel resistance.**

In a number of GC PDX models, significant differences in survival in response to paclitaxel compared to eribulin are observed; only those models are represented here. In these cases, paclitaxel resistance is not caused by increased *ABCB1* expression. (A) Uterine cancer PDX models: #343, uterine carcinosarcoma (paclitaxel vs eribulin *P* <0.005); #465, endometrioid endometrial carcinoma; (B) #333, Vulval adenocarcinoma arising from Paget’s disease; (C) #214 and #295, cervical adenocarcinoma; #271, cervical squamous cell carcinoma. Response to 3-week treatment regimens (indicated by black bar below x-axis) for eribulin and the standard OCS treatments cisplatin and paclitaxel (eribulin 1 mg/kg, 3 time/s weekly, IP; cisplatin 4 mg/kg, days 1, 8 and 18, IP; paclitaxel 25 mg/kg, twice weekly, IP). For the number of mice treated, time to progressive disease (PD), median time to harvest (TTH), and log-rank survival tests see Supplementary Table S8.

**Extended References**

1. Parslow AC, Parakh S, Lee FT, Gan HK, Scott AM. Antibody-Drug Conjugates for Cancer Therapy. *Biomedicines*. Jul 11 2016;4(3)doi:10.3390/biomedicines4030014

2. Dumontet C, Reichert JM, Senter PD, Lambert JM, Beck A. Antibody-drug conjugates come of age in oncology. *Nat Rev Drug Discov*. Aug 2023;22(8):641-661. doi:10.1038/s41573-023-00709-2

3. Elnakat H, Ratnam M. Distribution, functionality and gene regulation of folate receptor isoforms: implications in targeted therapy. *Adv Drug Deliv Rev*. Apr 29 2004;56(8):1067-84. doi:10.1016/j.addr.2004.01.001

4. Christoph DC, Asuncion BR, Hassan B, et al. Significance of folate receptor alpha and thymidylate synthase protein expression in patients with non-small-cell lung cancer treated with pemetrexed. *J Thorac Oncol*. Jan 2013;8(1):19-30. doi:10.1097/JTO.0b013e31827628ff

5. Toffoli G, Cernigoi C, Russo A, Gallo A, Bagnoli M, Boiocchi M. Overexpression of folate binding protein in ovarian cancers. *Int J Cancer*. Apr 22 1997;74(2):193-8. doi:10.1002/(sici)1097-0215(19970422)74:2<193::aid-ijc10>3.0.co;2-f

6. Parker N, Turk MJ, Westrick E, Lewis JD, Low PS, Leamon CP. Folate receptor expression in carcinomas and normal tissues determined by a quantitative radioligand binding assay. *Anal Biochem*. Mar 15 2005;338(2):284-93. doi:10.1016/j.ab.2004.12.026

7. Kalli KR, Oberg AL, Keeney GL, et al. Folate receptor alpha as a tumor target in epithelial ovarian cancer. *Gynecol Oncol*. Mar 2008;108(3):619-26. doi:10.1016/j.ygyno.2007.11.020

8. Ho GY, Vandenberg CJ, Lim R, et al. The microtubule inhibitor eribulin demonstrates efficacy in platinum-resistant and refractory high-grade serous ovarian cancer patient-derived xenograft models. *Ther Adv Med Oncol*. 2023;15:17588359231208674. doi:10.1177/17588359231208674

9. Shimizu T, Fujiwara Y, Yonemori K, et al. First-in-Human Phase 1 Study of MORAb-202, an Antibody-Drug Conjugate Comprising Farletuzumab Linked to Eribulin Mesylate, in Patients with Folate Receptor-alpha-Positive Advanced Solid Tumors. *Clin Cancer Res*. Jul 15 2021;27(14):3905-3915. doi:10.1158/1078-0432.CCR-20-4740

10. James RL, Sisserson T, Cai Z, et al. Development of an FRalpha Companion Diagnostic Immunohistochemical Assay for Mirvetuximab Soravtansine. *Arch Pathol Lab Med*. Jan 29 2024;doi:10.5858/arpa.2023-0149-OA

11. Furuuchi K, Rybinski K, Fulmer J, et al. Antibody-drug conjugate MORAb-202 exhibits long-lasting antitumor efficacy in TNBC PDx models. *Cancer Sci*. Jun 2021;112(6):2467-2480. doi:10.1111/cas.14898

12. Hasegawa K, Yagishita S, Shintani D, et al. Antitumor activity of farletuzumab ecteribulin in a panel of endometrial cancer patient-derived xenografts with four different molecular subtypes. *Annals of Oncology*. Oct 2023;34:S529-S529. doi:10.1016/j.annonc.2023.09.1964

13. Azumi M, Kusama K, Yoshie M, et al. Involvement of ferroptosis in eribulin-induced cytotoxicity in ovarian clear cell carcinoma. *Eur J Pharmacol*. May 15 2024;971:176544. doi:10.1016/j.ejphar.2024.176544

14. Patch AM, Christie EL, Etemadmoghadam D, et al. Whole-genome characterization of chemoresistant ovarian cancer. *Nature*. May 28 2015;521(7553):489-94. doi:10.1038/nature14410

15. Christie EL, Pattnaik S, Beach J, et al. Multiple ABCB1 transcriptional fusions in drug resistant high-grade serous ovarian and breast cancer. *Nat Commun*. Mar 20 2019;10(1):1295. doi:10.1038/s41467-019-09312-9

16. Robey RW, Pluchino KM, Hall MD, Fojo AT, Bates SE, Gottesman MM. Revisiting the role of ABC transporters in multidrug-resistant cancer. *Nat Rev Cancer*. Jul 2018;18(7):452-464. doi:10.1038/s41568-018-0005-8

17. Richardson DL, Moore KN, Vergote I, et al. Phase 1b study of mirvetuximab soravtansine, a folate receptor alpha (FRalpha)-targeting antibody-drug conjugate, in combination with carboplatin and bevacizumab in patients with platinum-sensitive ovarian cancer. *Gynecol Oncol*. Mar 5 2024;185:186-193. doi:10.1016/j.ygyno.2024.01.045

18. Moore KN, Oza AM, Colombo N, et al. Phase III, randomized trial of mirvetuximab soravtansine versus chemotherapy in patients with platinum-resistant ovarian cancer: primary analysis of FORWARD I. *Ann Oncol*. Jun 2021;32(6):757-765. doi:10.1016/j.annonc.2021.02.017

19. Yoshida T, Ozawa Y, Kimura T, et al. Eribulin mesilate suppresses experimental metastasis of breast cancer cells by reversing phenotype from epithelial-mesenchymal transition (EMT) to mesenchymal-epithelial transition (MET) states. *Br J Cancer*. Mar 18 2014;110(6):1497-505. doi:10.1038/bjc.2014.80

20. Ho GY, Kyran EL, Bedo J, et al. Epithelial-to-Mesenchymal Transition Supports Ovarian Carcinosarcoma Tumorigenesis and Confers Sensitivity to Microtubule Targeting with Eribulin. *Cancer Res*. Dec 2 2022;82(23):4457-4473. doi:10.1158/0008-5472.CAN-21-4012

21. Goto W, Kashiwagi S, Asano Y, et al. Eribulin Promotes Antitumor Immune Responses in Patients with Locally Advanced or Metastatic Breast Cancer. *Anticancer Res*. May 2018;38(5):2929-2938. doi:10.21873/anticanres.12541

22. Nesic K, Krais JJ, Wang Y, et al. BRCA1 secondary splice-site mutations drive exon-skipping and PARP inhibitor resistance. *Mol Cancer*. Aug 5 2024;23(1):158. doi:10.1186/s12943-024-02048-1

23. Wakefield MJ, Nesic K, Kondrashova O, Scott CL. Diverse mechanisms of PARP inhibitor resistance in ovarian cancer. *Biochim Biophys Acta Rev Cancer*. Dec 2019;1872(2):188307. doi:10.1016/j.bbcan.2019.08.002

24. Schindelin J, Arganda-Carreras I, Frise E, et al. Fiji: an open-source platform for biological-image analysis. *Nat Methods*. Jun 28 2012;9(7):676-82. doi:10.1038/nmeth.2019
