# Supplementary figures and images for: Farletuzumab ecteribulin and MORAb‐109, folate receptor alpha and mesothelin targeting antibody–drug conjugates, show activity in poor prognosis gynaecological cancer models
Source: Clin Transl Med. 2025 Mar 12;15(3):e70274. doi: 10.1002/ctm2.70274 (PMC11903193; doi:10.1002/ctm2.70274)

Figure S1

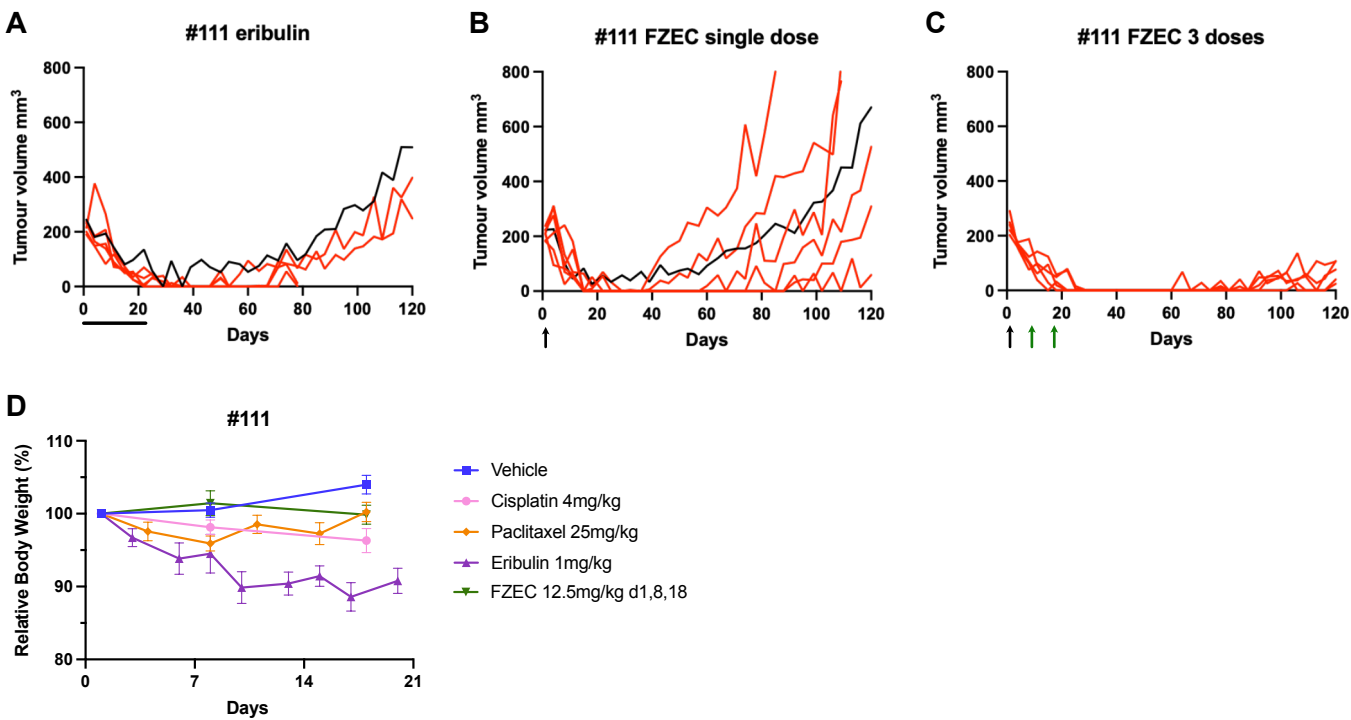

Figure S2

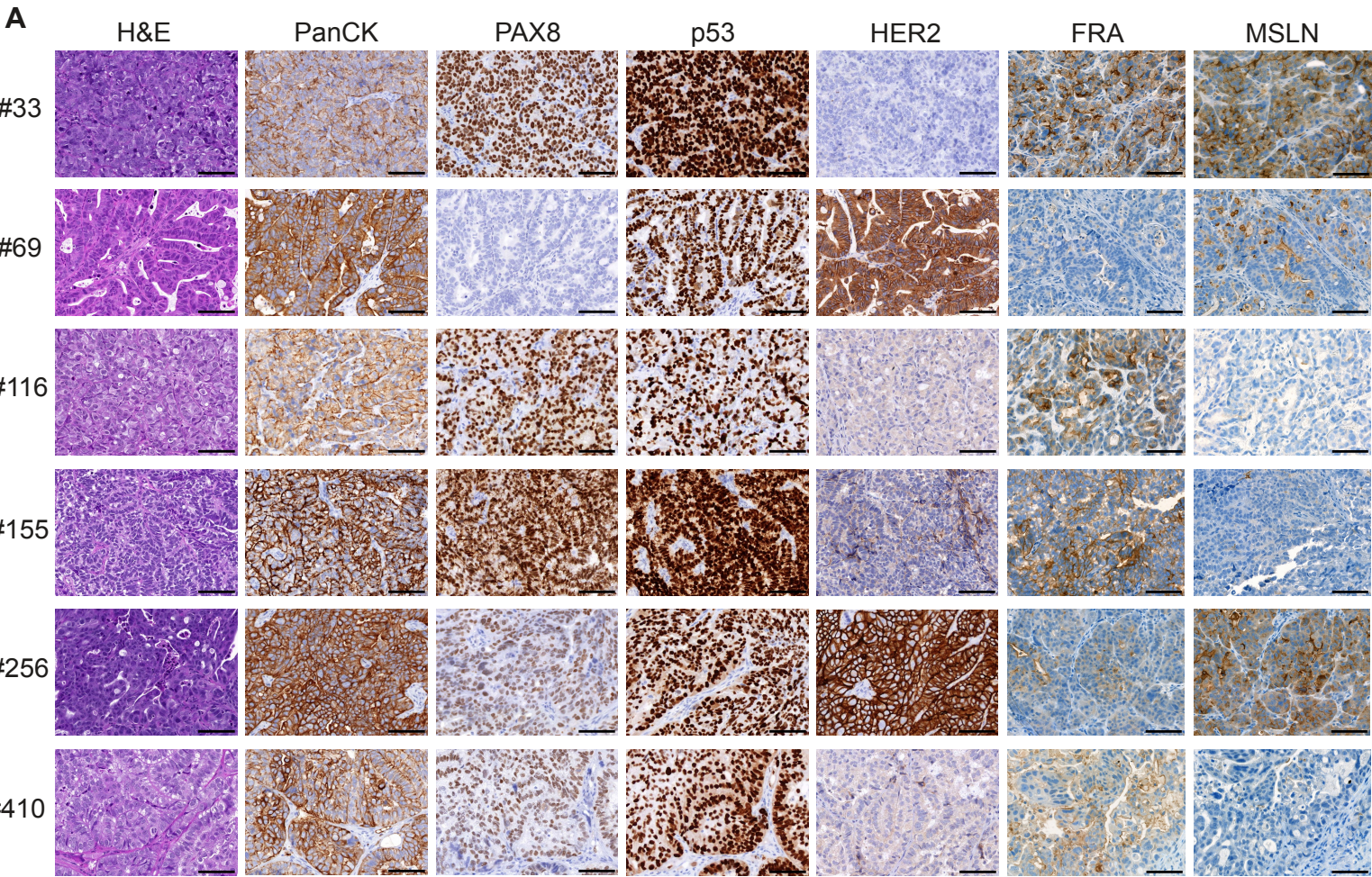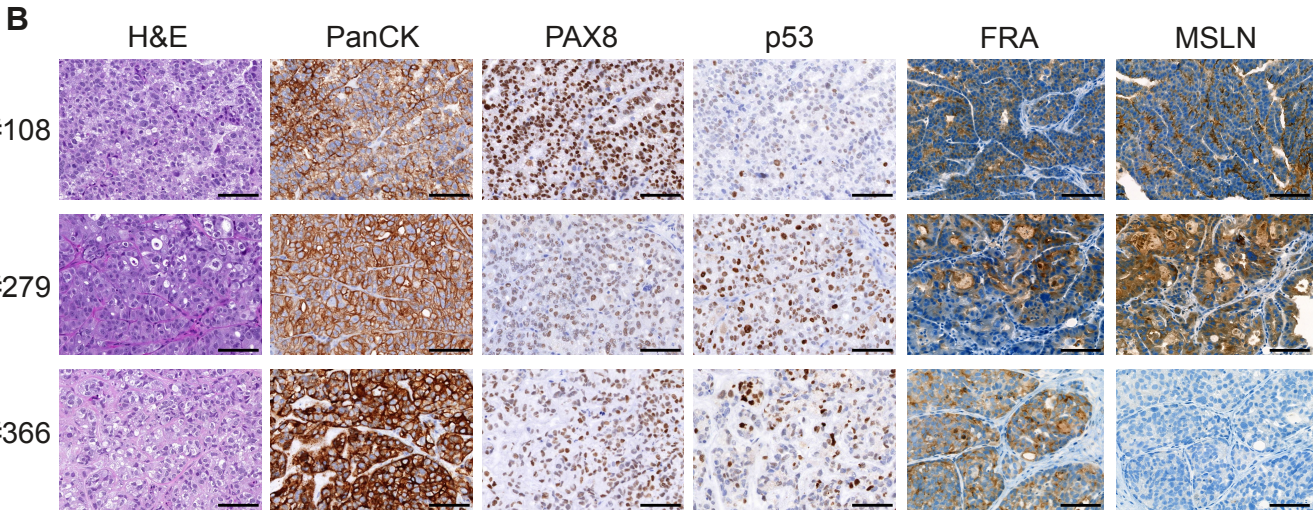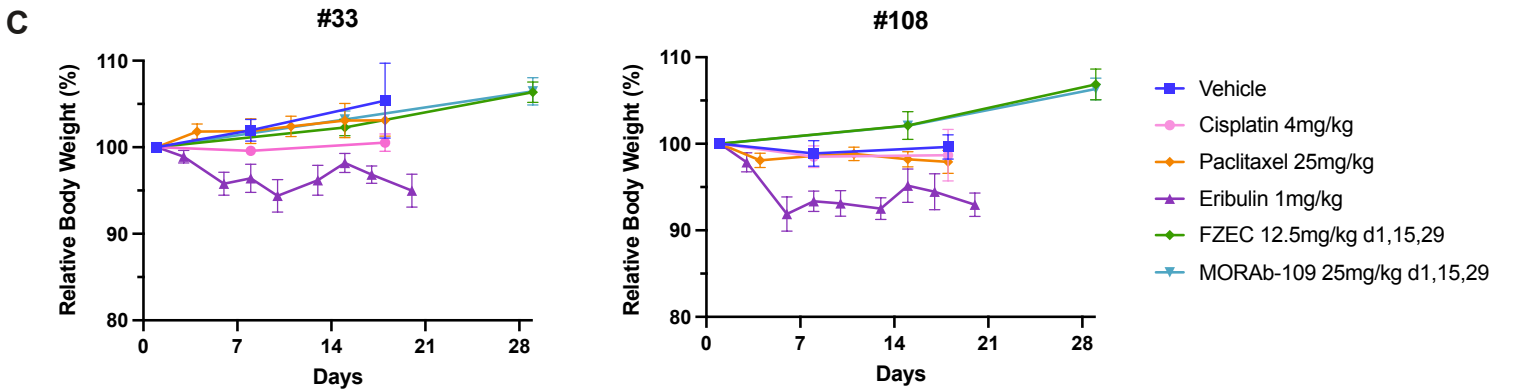

Figure S3

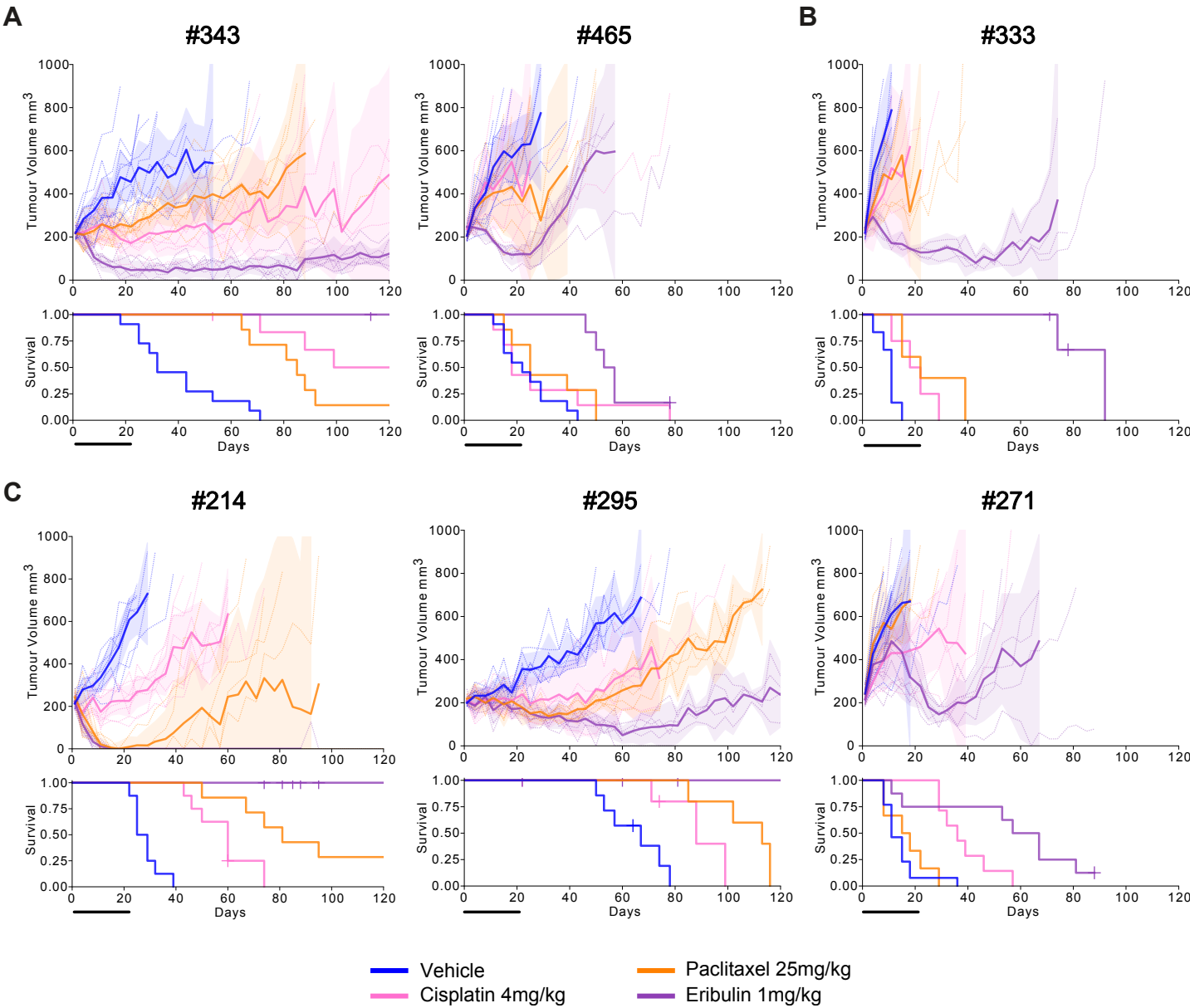

Supplement: Supplementary file 2 — Supporting Information [file CTM2-15-e70274-s001.pdf]
